# The plasma protein profile of left ventricular diastolic function

**DOI:** 10.1038/s41598-026-69505-3

**Published:** 2026-09-02

**Authors:** Elias Eerola, Lars Lind, Andrei Malinovschi

**Affiliations:** 1 Department of Medical Sciences, Clinical Physiology, Uppsala University, Uppsala, Sweden; 2 Department of Medical Sciences, Cardiology, Uppsala University, 751 85 Uppsala, Sweden; 3 Department of Medical Sciences, Clinical Epidemiology, Uppsala University, Uppsala, Sweden

**Keywords:** Diastolic function, Diastolic dysfunction, Heart failure, Echocardiography, Proteomics, Biomarkers, Cardiology, Diseases, Medical research

## Abstract

We aimed to characterize associations between plasma proteins and indices of left ventricular (LV) diastolic function in two population-based studies. Eighty-six cardiovascular-related plasma proteins measured by proximity extension assay (PEA) and echocardiography were assessed in the population-based studies PIVUS (*n* = 1,016; all aged 70) and POEM (*n* = 502; all aged 50). PIVUS served as the discovery cohort, and the Benjamini-Hochberg procedure was applied to control the false-discovery rate (FDR) < 0.05. POEM was used as the replication cohort. The protein panel was related to diastolic echocardiographic parameters. A larger protein panel of 1,322 proteins was examined only in POEM. Seven of 86 proteins showed replicated associations to the E/A ratio: CD40 ligand (CD40LG), proto-oncogene tyrosine-protein kinase Src (SRC), heat shock protein beta-1 (HSPB1), epidermal growth factor (EGF), TNF superfamily member 14 (TNFSF14), vascular endothelial growth factor D (VEGFD), and endothelial cell-specific molecule 1 (ESM1). Using the larger protein panel in POEM, 81 proteins showed significant associations with the E/A ratio, and five showed significant associations with LA diameter. Pathways linked to LV diastolic filling pattern were signal transduction, metabolic, disease and immune system pathways, enhancing the understanding of LV diastolic function and offering potential for future drug development.

**Supplementary Information:**

The online version contains supplementary material available at https://doi.org/10.1038/s41598-026-69505-3.

## Introduction

Heart failure (HF) with preserved ejection fraction (HFpEF) carries a large and significant global burden of disease, comprising approximately 50% of HF patients and roughly half of HF-related hospitalisations^1^. However, the pathophysiology of HFpEF remains poorly understood.

LV diastolic function can be evaluated by transthoracic echocardiography (TTE). Recently published guidelines by the British Society of Echocardiography highlight key measurements of diastolic function; E/e’ – the ratio between the early transmitral inflow (E) and the velocity of the mitral annulus measured by tissue Doppler (e’), TR velocity – the maximum velocity of the tricuspid regurgitation, and LA volume – the measured volume of the left atrium^2^. The relationship between early and late diastolic filling (E/A ratio) and isovolumetric relaxation time (IVRT) can be used as supplementary measures of diastolic function. A lower E/A ratio and an increase in IVRT indicate a relaxation disturbance of the myocardium (in the absence of elevated filling pressures)^2^.

A previous study^3^ investigated the relationship between 248 inflammatory proteins, using PEA and common HFpEF comorbidities (hypertension, obesity, and diabetes mellitus) in 228 HFpEF patients. Comorbidity burden was linked to systemic inflammation and abnormal cardiac function, and inflammatory proteins were upregulated in patients with HFpEF compared to controls. Furthermore, inflammatory proteins were associated with impaired diastolic parameters on TTE supporting the “comorbidity-inflammation” paradigm in HFpEF. Another study by Regan et al.^4^ assessed 459 proteins in HFpEF patients and validated externally their findings on 29 proteins associated with HFpEF. These biomarkers represented fibrosis, inflammation, cardiac remodelling, angiogenesis, and metabolism markers. The study also found links between some of the proteins and the prognosis of patients (hospitalisation and all-cause mortality).

A few studies have compared how plasma protein levels differ concerning the ejection fraction (EF). Adamo et al.^5^ investigated how plasma protein levels differ in HFpEF, HFrEF, and heart failure patients with mildly reduced EF (HFmrEF). In the HFpEF group, innate immunity-related proteins and a decrease in humoral immunity-related proteins were relevant. Another study, conducted in a broadly similar manner in patients with diabetes mellitus, found similar plasma protein levels in HFmrEF and HFpEF^6^. Finally, Andrzejczyk et al.^7^ investigated proteomic patterns in HF by comparing plasma protein levels of 4210 proteins in healthy adults, HFpEF, and HFrEF of ischaemic origin. The authors found that the plasma levels of two proteins, NELL2 (Neural EGFL-Like 2) and SLITRK6 (SLIT And NTRK Like Family Member 6), were lower in the HFpEF group compared to the other groups.

In population-based studies, few studies assessed the relation between LV diastolic dysfunction and circulating proteins. In a Belgian population-based study^8^, 575 participants (18% having diastolic dysfunction) underwent proteomic profiling by PEA, and TTE. The authors concluded that pathways related to inflammation, renal function, extracellular matrix remodelling, and angiogenesis were related to early-stage HF. The study also found specific proteins linked to diastolic dysfunction. However, the results were not externally validated in an independent cohort.

### Study aim

Although plasma proteomics has been explored in HFpEF, few population-based studies have assessed its association with indices of LV diastolic function. We conducted the first population-based investigation linking the plasma proteome to LV diastolic TTE parameters to fulfill this aim and validated our findings in an independent cohort.

## Methods

The Prospective Investigation of Vasculature in Uppsala Seniors (PIVUS) study^9^ was a population-based study with a major aim of investigating whether measurement of endothelial function would improve cardiovascular risk prediction. A total of 1,016 individuals, all 70 years of age and 50% women, were randomly selected from a population register in Uppsala County. All selected individuals underwent a series of investigations between 2001 and 2004, including TTE and proteomic profiling. All participants provided written informed consent prior to inclusion.

The Prospective Investigation of Obesity, Energy and Metabolism (POEM) study^9^ was a population-based study with the primary objective of investigating the links between cardiovascular disease (CVD) and obesity. In a similar manner, individuals were randomly selected from the population register in Uppsala County. The study included 502 50-year-olds, of which 50% were females. The individuals were examined between 2010 and 2016 and underwent a series of tests including TTE and proteomic profiling. All participants provided written informed consent prior to inclusion.

In both PIVUS and POEM, 92 plasma proteins were measured by PEA with the Cardiovascular Panel CVD-1 (Olink Proteomics AB). Proteins where < 70% of measurements were under the limit of detection (LOD) were excluded from the analysis. Of the 92 proteins, 6 had a call rate < 70%, leaving 86 proteins to be analysed.

Principal investigator Dr. Lind performed all TTE investigations in both PIVUS and POEM using an Acuson XP124 cardiac ultrasound unit (Acuson, California, USA) and a 2.5-MHz transducer. TTE included assessment of systolic function and geometry; however, for the purposes of this study, only the diastolic measurements were analysed: E/A ratio, IVRT, and LA diameter.

**Fig. 1:**
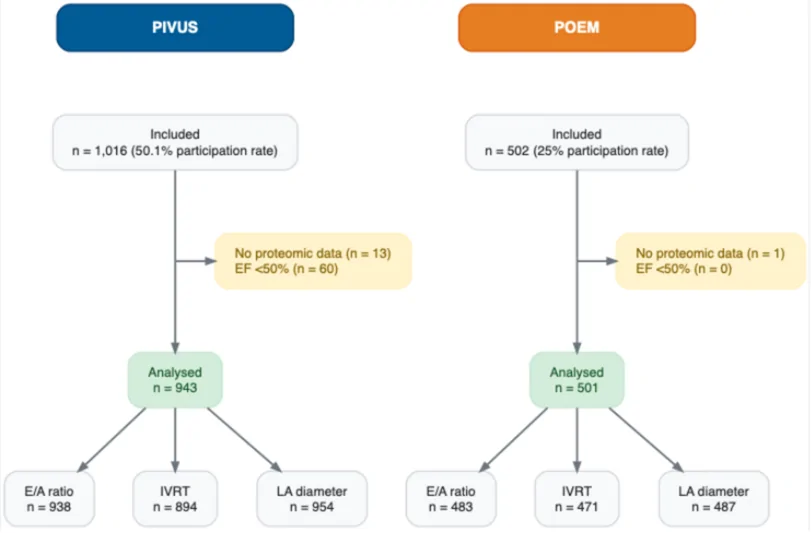
STROBE flow chart. PIVUS = The Prospective Investigation of Vasculature in Uppsala Seniors. POEM = The Prospective Investigation of Obesity, Energy and Metabolism.

Participants with left ventricular ejection fraction (LVEF) < 50% were excluded from the analysis to avoid pseudonormalisation of the E/A ratio and IVRT. Out of the 1,016 participants in PIVUS, 1,003 had proteomic measurements. Sixty participants were excluded due to EF < 50%. In POEM, all participants had an EF > 50%; only one was excluded due to lack of proteomic data. A STROBE flow chart is displayed in Fig. 1.

The diastolic indices and proteins levels were inverse-rank normalised to obtain normally distributed variables where both the exposure and the outcome were on the same z-scale.

Linear regression models were used to relate all 86 proteins one by one to each of the three LV diastolic function variables in both PIVUS and POEM. In the first set of models, we adjusted for age and sex only. In the second set of models, we adjusted for the traditional risk factors systolic blood pressure (BP), LDL- and HDL-cholesterol, diabetes, BMI, and smoking.

The proteins that showed a significant relation to one or more of the three TTE parameters in the PIVUS study were tested for replication in the POEM study. In the replication phase, the Benjamini-Hochberg procedure was applied to control the FDR < 0.05.

Additionally, an Olink Explore 1536 panel (Olink Proteomics AB) consisting of around 1,500 proteins was analysed in the POEM study. Of those, 1,322 proteins showed > 75% of the observations > LOD and were used in the extended analysis. The proteins were compared to the same diastolic TTE parameters as the 86 proteins. These results were not replicated. Bonferroni correction was used instead of Benjamini-Hochberg correction to decrease the risk of findings by chance (false positives). The proteins that passed the Bonferroni corrected *p*-value (0.000038) for model 1 and *p* < 0.05 for model 2 were considered significant.

Stata 16.1 (StataCorp, College Station, TX) was used for statistical analysis. Data visualisation was performed using R Statistical Software.

Olink Insight (Olink Proteomics AB) was used to analyse protein associations and map them to their underlying biological processes.

ChatGPT (OpenAI, 2025) was used for language editing. The author takes full responsibility for the content of the paper.

The investigation conforms to the principles of the Declaration of Helsinki, and all methods were performed in accordance with relevant guidelines and regulations. The Regional Ethics Committee in Uppsala, Sweden, approved the studies: Dnr 00/419 and 2011/045 (PIVUS) and Dnr 2009/057 (POEM). All participants provided written informed consent prior to inclusion.

## Results

**Fig. 2:**
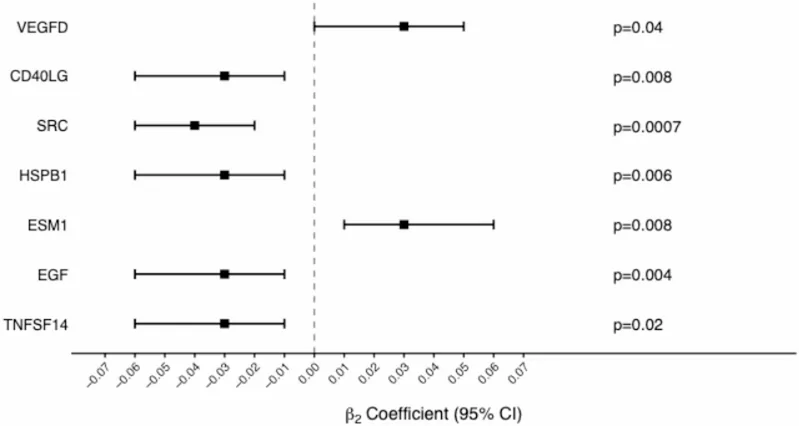
Forest plot of proteins associated with E/A that passed FDR-adjustment in PIVUS and could be replicated in POEM. VEGFD = Vascular endothelial growth factor D; CD40LG = CD40 ligand; SRC = Proto-oncogene tyrosine-protein kinase Src; HSPB1 = Heat shock protein beta-1; ESM1 = Endothelial cell-specific molecule 1; EGF = Epidermal growth factor; TNFSF14 = TNF superfamily member 14. Beta 2 = model adjusted for risk factors.

Participant characteristics in PIVUS and POEM are shown in Table 1. Of the 86 plasma proteins evaluated in PIVUS, 19 were significantly associated with the E/A ratio. In the POEM cohort, 15 of these 19 proteins remained significant after adjustment for age and sex, and seven retained significance after further adjustment for cardiovascular risk factors. These seven replicated proteins are listed in Fig. 2.

**Table 1:** Participant characteristics in PIVUS and POEM.

Variable	The Prospective Investigation of Vasculature in Uppsala Seniors (PIVUS)		The Prospective Investigation of Obesity, Energy and Metabolism (POEM)		*p*
	*n* analysed	Mean [SD] or %	*n* analysed	Mean [SD] or %	
Age *years*	1,016	70*	502	50*	-
Female sex %	1,016	50.2%	502	49.6%	NS
BMI *kg/m*^*2*^	1,016	27.0 [4.3]	502	26.5 [4.3]	NS
Current smoking *%*	1,015	10.5%	499	9.8%	NS
LDL *mmol/L*	1,011	3.3 [0.88]	502	3.4 [0.85]	NS
Systolic BP *mmHg*	1,012	149.6 [22.7]	502	125.6 [16.4]	0.001
Diastolic BP *mmHg*	1,012	78.7 [10.2]	502	77.0 [10.2]	NS
Diabetes *%*	1,010	8.7%	502	2.8%	0.001
E/A	938	0.96 [0.28]	483	1.5 [0.44]	0.001
IVRT *ms*	894	121.3 [21.1]	471	88.0 [13.3]	0.001
LA diameter *mm*	954	39.3 [6.7]	487	37.7 [4.6]	0.01

BMI = body mass index; LDL = low density lipoprotein; BP = blood pressure; NS = not significant (*p* > 0.05).

*All participants in PIVUS were 70 years old, and in POEM, 50 years old.

15 proteins out of the 86 screened in PIVUS were significantly associated with IVRT, but none remained significant after adjustment for age and sex in POEM. Four proteins out of the 86 screened in PIVUS were significantly associated with the LA diameter; all four remained significant after adjustment for age and sex in POEM, but none remained significant after adjustment for cardiovascular risk factors in POEM.

In the larger protein panel screened only in the POEM cohort, 81 proteins were significantly associated with the E/A ratio after Bonferroni correction and adjustment for age, sex, and cardiovascular risk factors. The proteins are displayed in Table 2.

**Table 2:** Proteins that passed the Bonferroni corrected *p* < (0.000038) and *p* < 0.05 for E/A ratio in POEM.

Gene name	β1	95% CI	*p* model 1*	β2	95% CI	*p* model 2**	Gene name	β1	95% CI	*p* model 1*	β2	95% CI	*p* model 2**
COMT	−0.22	[−0.31, −0.12]	0.0000056	−0.17	[−0.27, −0.08]	0.0003567	SRC	−0.19	[−0.28, −0.10]	0.0000377	−0.17	[−0.25, −0.08]	0.0003154
PAG1	−0.21	[−0.30, −0.12]	0.0000045	−0.16	[−0.26, −0.07]	0.0007713	MVK	−0.24	[−0.33, −0.14]	0.0000008	−0.16	[−0.26, −0.05]	0.0030498
USP8	−0.22	[−0.31, −0.13]	0.0000027	−0.19	[−0.28, −0.10]	0.0000694	TRAF2	−0.21	[−0.30, −0.12]	0.0000071	−0.17	[−0.26, −0.08]	0.0001561
PRKAR1A	−0.20	[−0.29, −0.11]	0.0000137	−0.18	[−0.27, −0.09]	0.0001310	TRIM21	−0.21	[−0.31, −0.12]	0.0000056	−0.17	[−0.26, −0.08]	0.0003430
PPP1R2	−0.20	[−0.29, −0.11]	0.0000164	−0.16	[−0.25, −0.07]	0.0007041	NFATC1	−0.19	[−0.28, −0.10]	0.0000320	−0.16	[−0.25, −0.07]	0.0006242
NADK	−0.25	[−0.34, −0.16]	0.0000000	−0.20	[−0.29, −0.11]	0.0000242	EGLN1	−0.20	[−0.29, −0.11]	0.0000198	−0.14	[−0.23, −0.04]	0.0055270
AHCY	−0.19	[−0.28, −0.10]	0.0000300	−0.14	[−0.24, −0.05]	0.0019450	HEXIM1	−0.21	[−0.31, −0.12]	0.0000074	−0.16	[−0.25, −0.07]	0.0007055
CD69	−0.20	[−0.30, −0.11]	0.0000198	−0.18	[−0.27, −0.09]	0.0001159	NUDC	−0.21	[−0.30, −0.12]	0.0000095	−0.16	[−0.26, −0.07]	0.0007003
TYMP	−0.19	[−0.28, −0.11]	0.0000207	−0.15	[−0.24, −0.06]	0.0008524	EIF4G1	−0.24	[−0.34, −0.15]	0.0000004	−0.20	[−0.29, −0.10]	0.0000689
LDLR	−0.21	[−0.30, −0.12]	0.0000073	−0.20	[−0.31, −0.08]	0.0007538	DNAJA2	−0.23	[−0.32, −0.14]	0.0000006	−0.20	[−0.29, −0.11]	0.0000300
CES1	−0.19	[−0.28, −0.11]	0.0000203	−0.11	[−0.20, −0.01]	0.0247887	PPP1R9B	−0.23	[−0.32, −0.14]	0.0000011	−0.20	[−0.29, −0.11]	0.0000215
PLAT	−0.27	[−0.36, −0.18]	0.0000000	−0.19	[−0.30, −0.08]	0.0005769	PRDX5	−0.24	[−0.33, −0.15]	0.0000002	−0.20	[−0.29, −0.11]	0.0000098
CORO1A	−0.31	[−0.40, −0.22]	0.0000000	−0.26	[−0.35, −0.16]	0.0000003	HCLS1	−0.23	[−0.33, −0.14]	0.0000010	−0.19	[−0.28, −0.09]	0.0000896
GUSB	−0.21	[−0.31, −0.12]	0.0000095	−0.15	[−0.25, −0.04]	0.0057889	PLA2G4A	−0.20	[−0.29, −0.11]	0.0000176	−0.19	[−0.28, −0.10]	0.0000761
ACP5	−0.23	[−0.33, −0.13]	0.0000038	−0.20	[−0.30, −0.10]	0.0000533	SIGLEC1	−0.19	[−0.28, −0.10]	0.0000207	−0.12	[−0.22, −0.03]	0.0096477
PPIB	−0.21	[−0.30, −0.12]	0.0000041	−0.18	[−0.27, −0.10]	0.0000362	GLOD4	−0.20	[−0.30, −0.11]	0.0000356	−0.16	[−0.26, −0.07]	0.0008287
PRCP	−0.22	[−0.31, −0.12]	0.0000079	−0.16	[−0.26, −0.06]	0.0022167	HSPA1A	−0.19	[−0.28, −0.10]	0.0000362	−0.13	[−0.23, −0.04]	0.0055261
SSC5D	−0.19	[−0.28, −0.10]	0.0000311	−0.13	[−0.23, −0.04]	0.0060789	SCGB3A2	0.20	[0.11, 0.29]	0.0000200	0.13	[0.03, 0.23]	0.0138614
RARRES2	−0.26	[−0.34, −0.17]	0.0000000	−0.19	[−0.29, −0.09]	0.0001627	PDLIM7	−0.20	[−0.29, −0.11]	0.0000186	−0.17	[−0.26, −0.08]	0.0003216
SELP	−0.19	[−0.28, −0.10]	0.0000371	−0.18	[−0.26, −0.09]	0.0000844	TPP1	−0.20	[−0.29, −0.11]	0.0000127	−0.15	[−0.24, −0.05]	0.0026398
C2	−0.29	[−0.37, −0.20]	0.0000000	−0.25	[−0.34, −0.16]	0.0000001	DAG1	−0.24	[−0.33, −0.15]	0.0000005	−0.18	[−0.28, −0.09]	0.0002179
RRM2B	−0.21	[−0.30, −0.12]	0.0000054	−0.15	[−0.24, −0.06]	0.0016689	MPIG6B	−0.21	[−0.30, −0.12]	0.0000093	−0.18	[−0.27, −0.09]	0.0000618
MAVS	−0.20	[−0.30, −0.11]	0.0000148	−0.17	[−0.26, −0.08]	0.0001683	TDRKH	−0.21	[−0.30, −0.12]	0.0000045	−0.17	[−0.25, −0.08]	0.0002454
GFER	−0.21	[−0.31, −0.12]	0.0000048	−0.16	[−0.26, −0.07]	0.0009518	DARS1	−0.20	[−0.29, −0.11]	0.0000351	−0.18	[−0.27, −0.09]	0.0001957
MPI	−0.24	[−0.33, −0.15]	0.0000003	−0.21	[−0.29, −0.12]	0.0000084	PTPN1	−0.22	[−0.31, −0.13]	0.0000037	−0.19	[−0.28, −0.10]	0.0000459
LAT2	−0.21	[−0.30, −0.12]	0.0000105	−0.17	[−0.26, −0.07]	0.0004119	CRADD	−0.20	[−0.30, −0.11]	0.0000256	−0.17	[−0.27, −0.08]	0.0003181
KRT18	−0.19	[−0.28, −0.10]	0.0000697	−0.12	[−0.22, −0.02]	0.0211867	WWP2	−0.20	[−0.29, −0.11]	0.0000202	−0.14	[−0.23, −0.04]	0.0043299
ERBIN	−0.22	[−0.31, −0.13]	0.0000022	−0.19	[−0.29, −0.10]	0.0000412	FKBP4	−0.21	[−0.30, −0.12]	0.0000137	−0.14	[−0.24, −0.04]	0.0051901
APBB1IP	−0.23	[−0.32, −0.14]	0.0000007	−0.14	[−0.24, −0.04]	0.0056662	PSME1	−0.21	[−0.31, −0.11]	0.0000307	−0.16	[−0.26, −0.06]	0.0017739
SIRT2	−0.24	[−0.33, −0.15]	0.0000002	−0.21	[−0.30, −0.11]	0.0000138	PLA2G10	0.20	[0.11, 0.29]	0.0000182	0.15	[0.05, 0.24]	0.0030872
DPY30	−0.21	[−0.30, −0.12]	0.0000047	−0.14	[−0.24, −0.04]	0.0046853	CCL19	−0.21	[−0.30, −0.12]	0.0000043	−0.16	[−0.25, −0.07]	0.0005951
CASP8	−0.22	[−0.32, −0.13]	0.0000032	−0.18	[−0.28, −0.09]	0.0001284	LBR	−0.26	[−0.35, −0.17]	0.0000000	−0.20	[−0.29, −0.10]	0.0000622
RTN4R	−0.20	[−0.29, −0.11]	0.0000157	−0.12	[−0.22, −0.02]	0.0143637	ENO1	−0.20	[−0.29, −0.11]	0.0000205	−0.15	[−0.24, −0.06]	0.0008826
DAB2	−0.19	[−0.28, −0.10]	0.0000353	−0.15	[−0.24, −0.06]	0.0010664	FABP5	−0.20	[−0.29, −0.11]	0.0000100	−0.13	[−0.23, −0.04]	0.0063781
METAP2	−0.23	[−0.32, −0.14]	0.0000017	−0.18	[−0.27, −0.08]	0.0005550	CLEC1B	−0.20	[−0.29, −0.12]	0.0000072	−0.20	[−0.28, −0.11]	0.0000107
FXN	−0.21	[−0.30, −0.12]	0.0000144	−0.16	[−0.25, −0.06]	0.0011987	INHBC	−0.23	[−0.32, −0.15]	0.0000002	−0.16	[−0.25, −0.06]	0.0011815
CDC37	−0.20	[−0.29, −0.11]	0.0000125	−0.17	[−0.26, −0.08]	0.0001663	TMSB10	−0.19	[−0.28, −0.10]	0.0000330	−0.13	[−0.23, −0.03]	0.0082221
ATOX1	−0.20	[−0.28, −0.11]	0.0000233	−0.15	[−0.24, −0.06]	0.0014710	ALDH1A1	−0.22	[−0.32, −0.12]	0.0000153	−0.14	[−0.25, −0.03]	0.0139830
CIAPIN1	−0.21	[−0.30, −0.11]	0.0000230	−0.17	[−0.26, −0.07]	0.0009375	ITGA5	−0.21	[−0.29, −0.12]	0.0000073	−0.14	[−0.23, −0.05]	0.0032106
MSRA	−0.22	[−0.32, −0.13]	0.0000026	−0.19	[−0.28, −0.09]	0.0001195	DBI	−0.25	[−0.34, −0.16]	0.0000001	−0.19	[−0.28, −0.09]	0.0001903
FURIN	−0.22	[−0.31, −0.13]	0.0000011	−0.12	[−0.23, −0.01]	0.0261223							

COMT = Catechol O-methyltransferase; PAG1 = Phosphoprotein associated with glycosphingolipid-enriched microdomains 1; USP8 = Ubiquitin carboxyl-terminal hydrolase 8; PRKAR1A = cAMP-dependent protein kinase type I-alpha regulatory subunit; PPP1R2 = Protein phosphatase inhibitor 2; NADK = NAD kinase; AHCY = Adenosylhomocysteinase; CD69 = Early activation antigen CD69; TYMP = Thymidine phosphorylase; LDLR = Low-density lipoprotein receptor; CES1 = Liver carboxylesterase 1; PLAT = Tissue-type plasminogen activator; CORO1A = Coronin-1 A; GUSB = Beta-glucuronidase; ACP5 = Tartrate-resistant acid phosphatase type 5; PPIB = Peptidyl-prolyl cis-trans isomerase B; PRCP = Lysosomal Pro-X carboxypeptidase; SSC5D = Soluble scavenger receptor cysteine-rich domain-containing protein SSC5D; RARRES2 = Retinoic acid receptor responder protein 2; SELP = P-selectin; C2 = Complement C2; RRM2B = Ribonucleoside-diphosphate reductase subunit M2 B; MAVS = Mitochondrial antiviral-signaling protein; GFER = FAD-linked sulfhydryl oxidase ALR; MPI = Mannose-6-phosphate isomerase; LAT2 = Linker for activation of T-cells family member 2; KRT18 = Keratin, type I cytoskeletal 18; ERBIN = Erbin; APBB1IP = Amyloid beta A4 precursor protein-binding family B member 1-interacting protein; SIRT2 = NAD-dependent protein deacetylase sirtuin-2; DPY30 = Protein dpy-30 homolog; CASP8 = Caspase-8; RTN4R = Reticulon-4 receptor; DAB2 = Disabled homolog 2; METAP2 = Methionine aminopeptidase 2; FXN = Frataxin, mitochondrial; CDC37 = Hsp90 co-chaperone Cdc37; ATOX1 = Copper transport protein ATOX1; CIAPIN1 = Anamorsin; MSRA = Mitochondrial peptide methionine sulfoxide reductase; FURIN = Furin; SRC = Proto-oncogene tyrosine-protein kinase Src; MVK = Mevalonate kinase; TRAF2 = TNF receptor-associated factor 2; TRIM21 = E3 ubiquitin-protein ligase TRIM21; NFATC1 = Nuclear factor of activated T-cells, cytoplasmic 1; EGLN1 = Egl nine homolog 1; HEXIM1 = Protein HEXIM1; NUDC = Nuclear migration protein nudC; EIF4G1 = Eukaryotic translation initiation factor 4 gamma 1; DNAJA2 = DnaJ homolog subfamily A member 2; PPP1R9B = Neurabin-2; PRDX5 = Peroxiredoxin-5, mitochondrial; HCLS1 = Hematopoietic lineage cell-specific protein; PLA2G4A = Cytosolic phospholipase A2; SIGLEC1 = Sialoadhesin; GLOD4 = Glyoxalase domain-containing protein 4; HSPA1A = Heat shock 70 kDa protein 1 A; SCGB3A2 = Secretoglobin family 3 A member 2; PDLIM7 = PDZ and LIM domain protein 7; TPP1 = Tripeptidyl-peptidase 1; DAG1 = Dystroglycan; MPIG6B = Megakaryocyte and platelet inhibitory receptor G6b; TDRKH = Tudor and KH domain-containing protein; DARS1 = Aspartate–tRNA ligase, cytoplasmic; PTPN1 = Tyrosine-protein phosphatase non-receptor type 1; CRADD = Death domain-containing protein CRADD; WWP2 = NEDD4-like E3 ubiquitin-protein ligase WWP2; FKBP4 = Peptidyl-prolyl cis-trans isomerase FKBP4; PSME1 = Proteasome activator complex subunit 1; PLA2G10 = Group 10 secretory phospholipase A2; CCL19 = C-C motif chemokine 19; LBR = Delta(14)-sterol reductase LBR; ENO1 = Alpha-enolase; FABP5 = Fatty acid-binding protein 5; CLEC1B = C-type lectin domain family 1 member B; INHBC = Inhibin beta C chain; TMSB10 = Thymosin beta-10; ALDH1A1 = Retinal dehydrogenase 1; ITGA5 = Integrin alpha-5; DBI = Acyl-CoA-binding protein.

*Model 1 = adjusted for age and sex.

**Model 2 = adjusted for risk factors.

No proteins associated with IVRT remained statistically significant after Bonferroni-correction in the POEM cohort. Five proteins, displayed in Table 3, were significantly associated with the LA diameter after Bonferroni-correction and correction for age, sex, and cardiovascular risk factors.

**Table 3:** Proteins that passed the Bonferroni corrected *p1* < (0.000038) and *p2* < 0.05 for LA diameter in POEM.

Gene name	β1	95% CI	*p* model 1*	β2	95% CI	*p* model 2**
KITLG	−0.18	[−0.26, −0.10]	0.00001	−0.1	[−0.17, −0.03]	0.009
ADAMTS15	0.26	[0.17, 0.34]	0.0000000	0.09	[0.01, 0.17]	0.0232918
AGER	−0.17	[−0.26, −0.09]	0.00003	−0.09	[−0.16, −0.01]	0.023
NCAN	−0.22	[−0.30, −0.14]	0.0000002	−0.08	[−0.16, −0.00]	0.0418459
NCAM2	−0.21	[−0.29, −0.13]	0.0000003	−0.10	[−0.18, −0.03]	0.0078025

KITLG = Kit ligand; ADAMTS15 = A disintegrin and metalloproteinase with thrombospondin motifs 15; AGER = Advanced glycosylation end product-specific receptor (RAGE); NCAN = Neurocan core protein; NCAM2 = Neural cell adhesion molecule 2.

*Model 1 = adjusted for age and sex.

**Model 2 = adjusted for risk factors.

Olink Insight was used to aid pathway mapping. Key pathways associated with the E/A ratio of the seven proteins from the discovery and replication parts of the study were signal transduction and immune system pathways. EGF, HSPB1, and SRC were related to signal transduction pathways, whereas TNFSF14, CD40L, and SRC were related to immune system pathways (with SRC appearing in both).

The predominant pathways for the 81 proteins from the broader protein panel in POEM were signal transduction, metabolism, disease, and immune system pathways. The pathways and associated proteins are displayed in Table 4.

**Table 4:** Proteins associated with different biological pathways, as identified by Olink Insight.

Pathway (*n* of genes)	Associated genes
**Signal transduction (25)**	CCL19, CASP8, WWP2, FURIN, PTPN1, USP8, FKBP4, PSME1, RTN4R, PAG1, NFATC1, ALDH1A1, SRC, ITGA5, PLA2G4A, EIF4G1, TRAF2, APBB1IP, ERBIN, NUDC, PLAT, PRKAR1A, FABP5, LBR, CDC37
**Metabolism (22)**	PLA2G10, COMT, MVK, PSME1, TYMP, ACP5, ALDH1A1, LDLR, NADK, GUSB, CES1, AHCY, PLA2G4A, RRM2B, ENO1, DBI, PRKAR1A, DARS1, FABP5, LBR, FXN, CIAPIN1
**Disease (20)**	CASP8, FURIN, COMT, FKBP4, PSME1, SRC, GUSB, PPIB, AHCY, TRAF2, DAG1, MAVS, APBB1IP, ERBIN, CORO1A, ENO1, HSPA1A, PRKAR1A, MPI, CDC37
**Immune system (19)**	CCL19, CASP8, PTPN1, TRIM21, PSME1, LAT2, PAG1, NFATC1, ATOX1, SRC, GUSB, C2, PRCP, EIF4G1, TRAF2, MAVS, SIGLEC1, HSPA1A, FABP5
**Metabolism of proteins (12)**	FURIN, USP8, PSME1, CES1, INHBC, EIF4G1, TRAF2, DAG1, MAVS, DARS1, MPI, MSRA

We screened for non-linear relationships by introducing a quadratic term for the protein in the models. After applying the same FDR-correction of the p-value and the same discovery/validation approach as performed in the linear analyses, no significant non-linear relationships were seen for any of the 86 proteins regarding LA or IVRT, but in the discovery cohort a significant non-linear term was seen for Renin regarding E/A ratio (*p* = 0.00001). Such inverted U-shaped relationship was also found in the validation cohort (*p* = 0.028) and is shown in a figure in the supplementary material (created by a restricted cubic spline function for renin with knots at 10, 50 and 90%). Thus, it seems as if both high and low levels of renin are associated with a low E/A-ratio.

## Discussion

We identified seven proteins whose associations with the E/A ratio were successfully replicated in an independent cohort. These proteins are involved in signal transduction and immune system mechanisms. No proteins could be validated to be associated with IVRT or LA diameter. Furthermore, we could identify an additional 81 proteins in an explorative analysis of a broader protein panel to be associated with E/A and five proteins related to LA diameter.

The PREHOSP-CHF study^10^ found that VEGFD levels, a protein involved in lymphangiogenesis and angiogenesis, were similar in HFpEF, HFmrEF, and HFrEF patients. In the HFpEF group, they found that VEGFD levels were linked to a higher risk of heart-failure hospitalisation in HFpEF. This contradicts the present study, which found a positive correlation between the E/A ratio and the same protein. CD40 ligand is a protein expressed on activated T-cells that is important for immune system function. To the authors’ knowledge, no study has linked CD40-ligand to diastolic dysfunction. One study concluded that patients with both acute and chronic HF had elevated levels of CD40L^11^. However, the LVEF levels were lower in the cited study compared to the LVEF of the participants in our study. Since elevated CD40L-levels are often seen in patients with hypertension and diabetes^12^, which are common comorbidities in HFpEF, this could serve as a possible mechanism of elevated CD40L-levels in diastolic dysfunction.

SRC, a protein essential in the downstream signalling of β-adrenergic receptors, was found by Takeishi et al. to be activated in the heart by acute myocardial stretch and chronic pressure overload in guinea pigs^13^. A 2023 animal study^14^ demonstrated that SRC-signalling mediates cardiac remodelling and fibrosis as a possible mechanism for diastolic dysfunction. Although these are animal data, these results align with the findings of our human observations. HSPB1, a stress-response chaperone, was found in the present study to be negatively correlated with the E/A ratio. The current pool of evidence indicates that HSPB1 is up-regulated in cellular stress states^15^. In the ULSAM cohort, 70-year-olds free from CVD were followed for incident events, including HF. In the HF-subgroup, HSPB1 was positively correlated with incident HF^16^. Another study found a link between HSPB1 levels and poor prognosis in chronic HF patients^17^. However, the mean LVEF in the latter study was lower than that of the participants in our study.

ESM1 or Endocan is synthesised by endothelial cells. Endocan levels have been shown to correlate with HF-related adverse events in patients with chronic HF^18^. The mean LVEF in the study was 36% but the study also included patients with preserved LVEF (> 50%). Although it is hard to make a direct comparison, the results seem slightly contradictory to the present study, where endocan levels were positively correlated with the E/A ratio. EGF receptor is involved in pathways related to cell survival and cell division. In a mouse model, EGFR-knockout mice were shown to develop contractile dysfunction and impaired diastolic relaxation^19^. Conversely, a study investigating hypertrophic cardiomyopathy (HCM) patients found that EGF receptor activation contributed to impaired relaxation^20^. The mouse model demonstrates a causal relationship between EGFR-signalling and diastolic dysfunction. In contrast, an opposite correlation was shown in a human study and in the present study, possibly highlighting a complex relationship of EGFR-signalling and diastolic function.

The pro-inflammatory cytokine TNFSF14/LIGHT was negatively correlated with the E/A ratio in the present study. Dahl et al. concluded that TNFSF14 has a role in the progression of chronic HF^21^. However, the study examined rats and tissue samples of human hearts with an LVEF < 35% waiting for transplantation. A recent mouse study^22^ found that TNFSF14 promotes atrial fibrillation and atrial fibrosis, a common comorbidity of diastolic dysfunction, highlighting a possible mechanism for the relationship to diastolic dysfunction. Thus, the association between LIGHT and diastolic function is not well established, as no human studies have investigated this specific connection.

In the discovery-only part of the present study a total of 81 proteins surpassed the Bonferroni adjusted threshold (*p* < 0.000038) for the E/A ratio. Five proteins were found to have a significant association with the LA diameter, which is notable. However, these findings are not explored further here because LA diameter alone is considered too nonspecific for the definitive assessment of diastolic function. Out of the 81 proteins, several have been linked to diastolic function in preclinical studies. Still, a few stood out as particularly relevant based on previous literature linking them to LV diastolic function in humans.

Füth et al. investigated the role of P-Selectin, a protein produced by activated platelets, regarding diastolic function. The authors found higher levels of P-selectin in patients with diastolic dysfunction, independent of diabetes mellitus or coronary artery disease^23^. The patients were evaluated with TTE and had a mean EF of 68%. Tissue-type plasminogen activator (tPA) is a protein that facilitates the breakdown of blood clots by converting plasminogen to plasmin. Winter et al. demonstrated that the tPA/PAI-1 complex independently predicts all-cause and cardiac mortality in HFpEF patients^24^. Another study found elevated levels of tPA along with other biomarkers of procoagulation, proposing that HFpEF is associated with a procoagulant state^25^. Adenosylhomocysteinase is the precursor of homocysteine. There have been several papers linking homocysteine levels to diastolic dysfunction. In a recent cohort of roughly 6700 patients, homocysteine levels were shown to contribute to HF, especially HFpEF^26^. Another study found elevated homocysteine levels in HFpEF patients compared to controls^27^.

Of the 81 proteins discovered in the Explorer panel, only one protein was found among the seven proteins from the discovery and replication part of the study, namely SRC. Comparing the results to the recently published UK Biobank study^28^ of proteomics we can conclude that out of the seven important proteins found in the discovery and replication part of the study, two proteins are found in the list of 683 proteins linked to HF: TNFSF14 and VEGFD. From the larger protein panel an additional 14 proteins can be found among the list of 683 proteins: DPY30, FKBP4, FURIN, ITGA5, LBR, PLAT, PRCP, PTPN1, RARRES2, SCGB3A2, SIGLEC1, TMSB10, TPP1, and TYMP.

To our knowledge, very few studies have been conducted in a similar manner as ours. Cauwenberghs et al.^8^ investigated the relationship between diastolic echocardiographic parameters and proteomics in a population-based cohort; however, their findings lacked an independent replication cohort. They measured a host of diastolic parameters including E/A ratio and LA volume, but not IVRT. Interestingly, they identified associations with a different set of proteins than those observed in our study.

Our study participants had an average IVRT of 121 ms in the cohort of 70-year-olds and 88 ms in the 50-year-olds. The average E/A ratios were 0.96 and 1.5, respectively. The higher E/A ratio and lower IVRT in the 50-year-olds are suggestive of less stiff hearts in the younger population and both sets of values are in keeping with expected values for respective ages^29,30^.

### Study strengths and limitations

Few studies have compared the above-mentioned proteins to LV diastolic function, which is a strength of the present study. Additional methodological strengths include validation in an external cohort. The same experienced clinician performed all TTE examinations, and protein profiling was performed with two different protein panels.

In the present study, significance was found only for the E/A ratio. This could be considered a limitation as we did not find the same relationship between IVRT and LA diameter. However, the E/A ratio is the most robust because it exhibits minimal interobserver variation and can be obtained even when image quality is suboptimal. Another weakness was that other diastolic TTE parameters, such as E/e’ and TR velocity, were not measured.

Since the study population consisted of 50- and 70-year-old volunteers, it is uncertain if the results apply to a broader age range. The population-based design can be regarded both as a weakness with regard to the relevance of our findings for HFpEF patients, but also as a strength, as these protein changes can be related to early signs of diastolic dysfunction.

## Conclusions

The present study is one of the few studies investigating the relationship of proteomics and LV diastolic function in a population-based cohort and externally validating the results in an external cohort. Key pathways linked to diastolic filling pattern were signal transduction, metabolic, and immune system pathways. Notably, we identified several proteins linked to diastolic function that previous studies have associated with diastolic dysfunction or HFpEF. These include proteins validated in the discovery and replication cohorts (Src, EGF) as well as proteins identified in our broader discovery panel (P-selectin, tPA, and AHCY). The study enhances understanding of the pathophysiology of diastolic dysfunction, offering potential for future drug development.

## Supplementary Information

Below is the link to the electronic supplementary material.


Supplementary Material 1



Supplementary Material 2


## Data Availability

A supplementary Excel file containing the results of all data analysis is attached. De-identified source data of protein values and echo variables are available upon reasonable request.
