# Supplementary material for: The plasma protein profile of left ventricular diastolic function
Source: Sci Rep. 2026 Sep 2;16:27502. doi: 10.1038/s41598-026-69505-3 (PMC13534439; doi:10.1038/s41598-026-69505-3)
Supplement: Supplementary file 1 [file 41598_2026_69505_MOESM2_ESM.pdf]

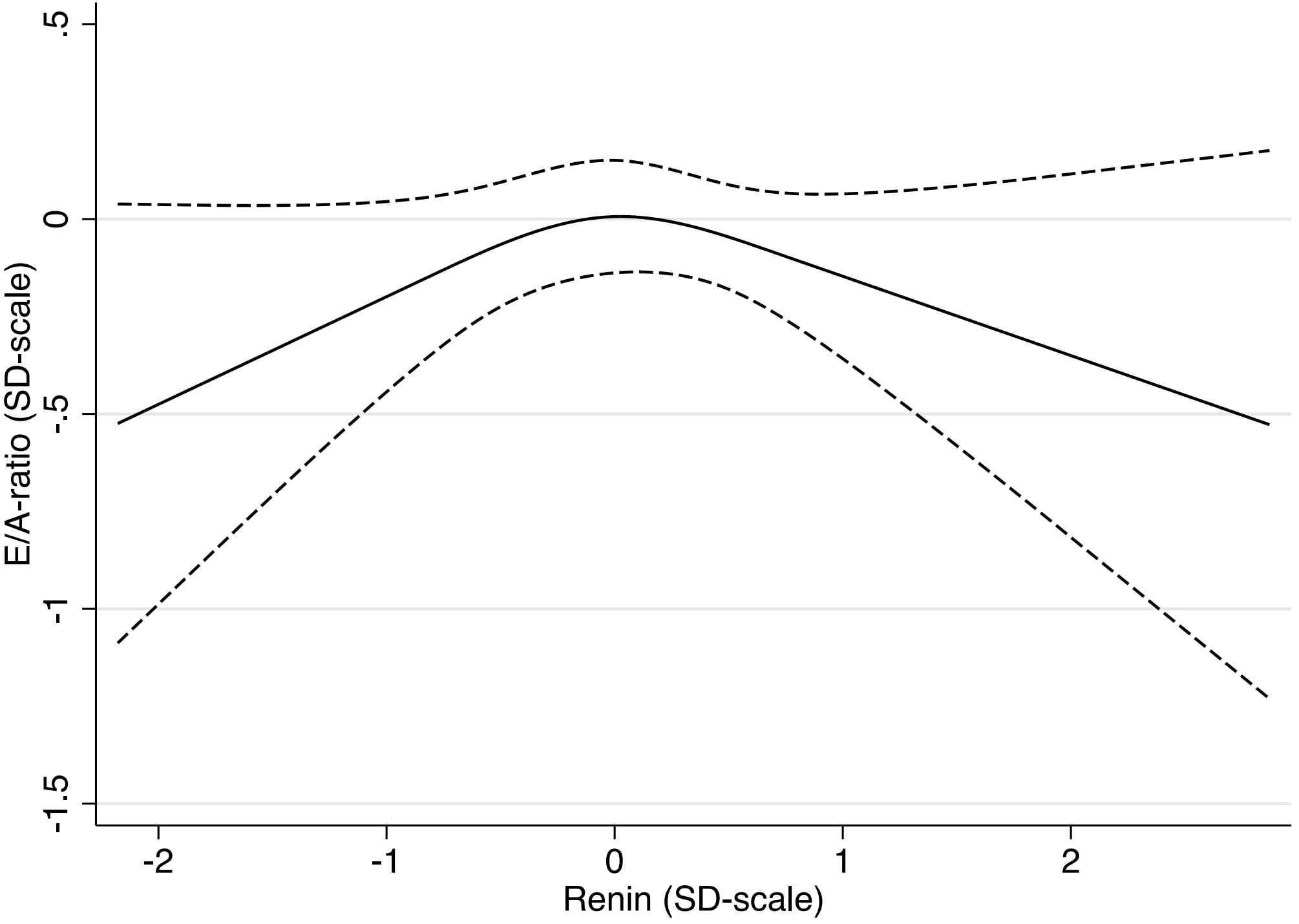

.5
0
-.5
-1
-1.5
E/A-ratio (SD-scale)
-2
-1
0
1
2
Renin (SD-scale)
